# Exposure to *Trypanosoma* parasites induces changes in the microbiome of the Chagas disease vector *Rhodnius prolixus*

**DOI:** 10.1186/s40168-022-01240-z

**Published:** 2022-03-10

**Authors:** Fanny E. Eberhard, Sven Klimpel, Alessandra A. Guarneri, Nicholas J. Tobias

**Affiliations:** 1 Institute for Ecology, Evolution and Diversity, Goethe University Frankfurt, Biologicum Campus Riedberg, Max-von-Laue-Str. 13, 60439 Frankfurt/Main, Germany; 2 LOEWE Centre for Translational Biodiversity Genomics (LOEWE TBG), Senckenberganlage 25, 60325 Frankfurt/Main, Germany; 3 Senckenberg Gesellschaft für Naturforschung, Senckenberg Biodiversity and Climate Research Centre, Senckenberganlage 25, 60325 Frankfurt/Main, Germany; 4 Vector Behaviour and Pathogen Interaction Group, Instituto René Rachou, Avenida Augusto de Lima,1715, Belo Horizonte, MG CEP 30190-009 Brazil

**Keywords:** Intestinal bacterial community, Triatominae, Host-parasite interaction, *Trypanosoma cruzi*, *Trypanosoma rangeli*, Secondary metabolites, Metagenomic shotgun sequencing

## Abstract

**Background:**

The causative agent of Chagas disease, *Trypanosoma cruzi*, and its nonpathogenic relative, *Trypanosoma rangeli*, are transmitted by haematophagous triatomines and undergo a crucial ontogenetic phase in the insect’s intestine. In the process, the parasites interfere with the host immune system as well as the microbiome present in the digestive tract potentially establishing an environment advantageous for development. However, the coherent interactions between host, pathogen and microbiota have not yet been elucidated in detail. We applied a metagenome shotgun sequencing approach to study the alterations in the microbiota of *Rhodnius prolixus*, a major vector of Chagas disease, after exposure to *T. cruzi* and *T. rangeli* focusing also on the functional capacities present in the intestinal microbiome of the insect.

**Results:**

The intestinal microbiota of *R. prolixus* was dominated by the bacterial orders *Enterobacterales*, *Corynebacteriales*, *Lactobacillales*, *Clostridiales* and *Chlamydiales*, whereas the latter conceivably originated from the blood used for pathogen exposure. The anterior and posterior midgut samples of the exposed insects showed a reduced overall number of organisms compared to the control group. However, we also found enriched bacterial groups after exposure to *T. cruzi* as well as *T rangeli*. While the relative abundance of *Enterobacterales* and *Corynebacteriales* decreased considerably, the *Lactobacillales*, mainly composed of the genus *Enterococcus*, developed as the most abundant taxonomic group. This applies in particular to vectors challenged with *T. rangeli* and at early timepoints after exposure to vectors challenged with *T. cruzi*. Furthermore, we were able to reconstruct four metagenome-assembled genomes from the intestinal samples and elucidate their unique metabolic functionalities within the triatomine microbiome, including the genome of a recently described insect symbiont, *Candidatus Symbiopectobacterium*, and the secondary metabolites producing bacteria *Kocuria* spp.

**Conclusions:**

Our results facilitate a deeper understanding of the processes that take place in the intestinal tract of triatomine vectors during colonisation by trypanosomal parasites and highlight the influential aspects of pathogen-microbiota interactions. In particular, the mostly unexplored metabolic capacities of the insect vector’s microbiome are clearer, underlining its role in the transmission of Chagas disease.

Video Abstract

**Supplementary Information:**

The online version contains supplementary material available at 10.1186/s40168-022-01240-z.

## Background

The microbial community inhabiting a eukaryotic organism is characterised by its bacterial, viral, fungal, algal and protozoan members and fulfils several fundamental functions including the support of host immunity, cold tolerance and manipulation of host behaviour [1–3]. In insects in particular, the provision of essential nutrients is also an important part of the microbiome-host relationship as well as the support of diet digestion and detoxification [4]. These interactions and the associated microbial composition are shaped by external influences such as food resources, geographic location and climate, landscape and a natural or laboratory environment [5–7]. Furthermore, the microbiome also influences the ability of insects to transmit pathogens and, therefore, to act as disease vectors. For instance, the mosquito *Anopheles gambiae* displays an increased susceptibility to *Plasmodium falciparum*, the etiological agent of malaria, when their natural microbiota is eradicated [8]. A reciprocal interaction of host microbiota and disease agents was also found in other protozoan pathogens developing in the gastrointestinal system of insects, such as *Leishmania*, which is transmitted by sand flies and causes leishmaniosis, or *Trypanosoma brucei*, the etiological agent of sleeping sickness transmitted by tsetse flies [9–12].

Another trypanosomal pathogen that undergoes an ontogenetic phase in the intestinal tract of insects is *Trypanosoma cruzi*. This protozoan parasite is harboured by haematophagous “kissing bugs” of the subfamily Triatominae (Hemiptera: Reduviidae) and causes the neglected tropical Chagas disease (American trypanosomiasis) in humans. It is estimated that 6 to 7 million people worldwide are affected by this disease, with severe medical, social and economic consequences. Most of them live in rural regions of Latin America, but due to emigration of population, Chagas disease has increasingly spread to urban areas and non-endemic countries with non-vectorial transmission routes [13]. In addition, some triatomine vectors, especially the cosmopolitan *Triatoma rubrofasciata*, also show a considerable potential for further geographical expansion [14–16]. Unlike in other blood-sucking insects, *T. cruzi* is not transmitted by the bite but by infectious faeces which are accidentally rubbed into the bite wound. Further transmission routes include oral consumption of contaminated food or beverages, congenital transmission and the transmission by infected blood products or organ transplantation [17–19]. *Trypanosoma rangeli*, a relative of *T. cruzi*, is transmitted through the infectious saliva of the triatomine vector during a blood meal but is not known to cause serious disease in humans. However, some vectors are impaired in their reproductive abilities and retarded in development by *T. rangeli* infection [20, 21]. Furthermore, the infection with trypanosomal parasites has further effects on the overall physiological state of the triatomine vector by interfering with its immune system and altering the inherent microbial community of its host [22, 23]. Vectors infected with *T. cruzi* show a considerably lower microbial diversity, presumably triggered by the insect’s immune reactions, which could be crucial for the parasite’s survival [24, 25]. An infection also promotes a changed abundance of distinct bacterial groups as it was shown for *Enterococcaceae*, *Burkholderiaceae*, *Enterobacterales* and the genus *Bacillus*. Since bacterial microorganisms are important suppliers of nutrients as well as secondary metabolites, this may also influence the chemical ecology prevalent in the insect’s intestinal system [23, 26, 27]. Secondary metabolites, which are not vital for the development and reproduction of an organism, but often provide an ecologically selective advantage, may play a role here. In this regard, they can support nutrient absorption and stress resistance but also increased virulence and defence mechanisms mediating interspecific relations and competition. For example, it has been shown that the pigment prodigiosin produced by the *Rhodnius prolixus*’ gut bacteria *Serratia marcescens* has a trypanolytic effect against *T. cruzi* [28]. Nevertheless, the specific interactions between vector, microbiota and pathogen which occur in the insect’s intestine and lead to the infestation of the vector remain unclear.

Here, we used metagenomic shotgun sequencing to characterise the intestinal microbiome of the triatomine vector *R. prolixus* and assess the changes occurring in the microbial community during exposure with the Chagas disease agent, *T. cruzi*, and its nonpathogenic relative *T. rangeli*. Special attention was paid to the metabolic capacities present in the detected bacteria, particularly with regard to nutrient supply and secondary metabolites potentially mediating the parasite infestation. To our knowledge, this the first comprehensive large-scale study on the microbiota of Chagas disease vectors using a whole metagenomic shotgun sequencing approach to elucidate the interlaced pathogen-vector-microbiome relationship.

## Methods

### Ethics statement

All experiments using living animals were performed in accordance with FIOCRUZ guidelines on animal experimentation, adhering to all Brazilian legislation regarding animal welfare. Protocols were based upon procedures set out by the Ministry of Science and Technology (CONCEA/MCT) associated with the American Association for Animal Science (AAAS), the Federation of European Laboratory Animal Science Associations (FELASA), the International Council for Animal Science (ICLAS) and the Association for Assessment and Accreditation of Laboratory Animal Care International (AAALAC). Experiments were approved by the Committee for Ethics in the Use of Animals, CEUA-FIOCRUZ, under the license number LW-8/17.

### Insect rearing and infection

*Rhodnius prolixus* nymphs used in this study were reared by the Vector Behaviour and Pathogen Interaction Group, FIOCRUZ, Belo Horizonte, Brazil. The insects were maintained at a temperature of 25 ± 1 °C, 60 ± 10% relative humidity and natural illumination and were fed monthly with citrated rabbit or sheep blood (10% v/v; Centro de Criação de Animais de Laboratório, CECAL, Fiocruz, Rio de Janeiro, Brazil) offered in an artificial feeder, and the chicken was anaesthetised with an intraperitoneal injection of a mixture of ketamine (20 mg/kg; Cristália, Brazil) and detomidine (0.3 mg/kg; Syntec, Brazil).

**Fig. 1:**
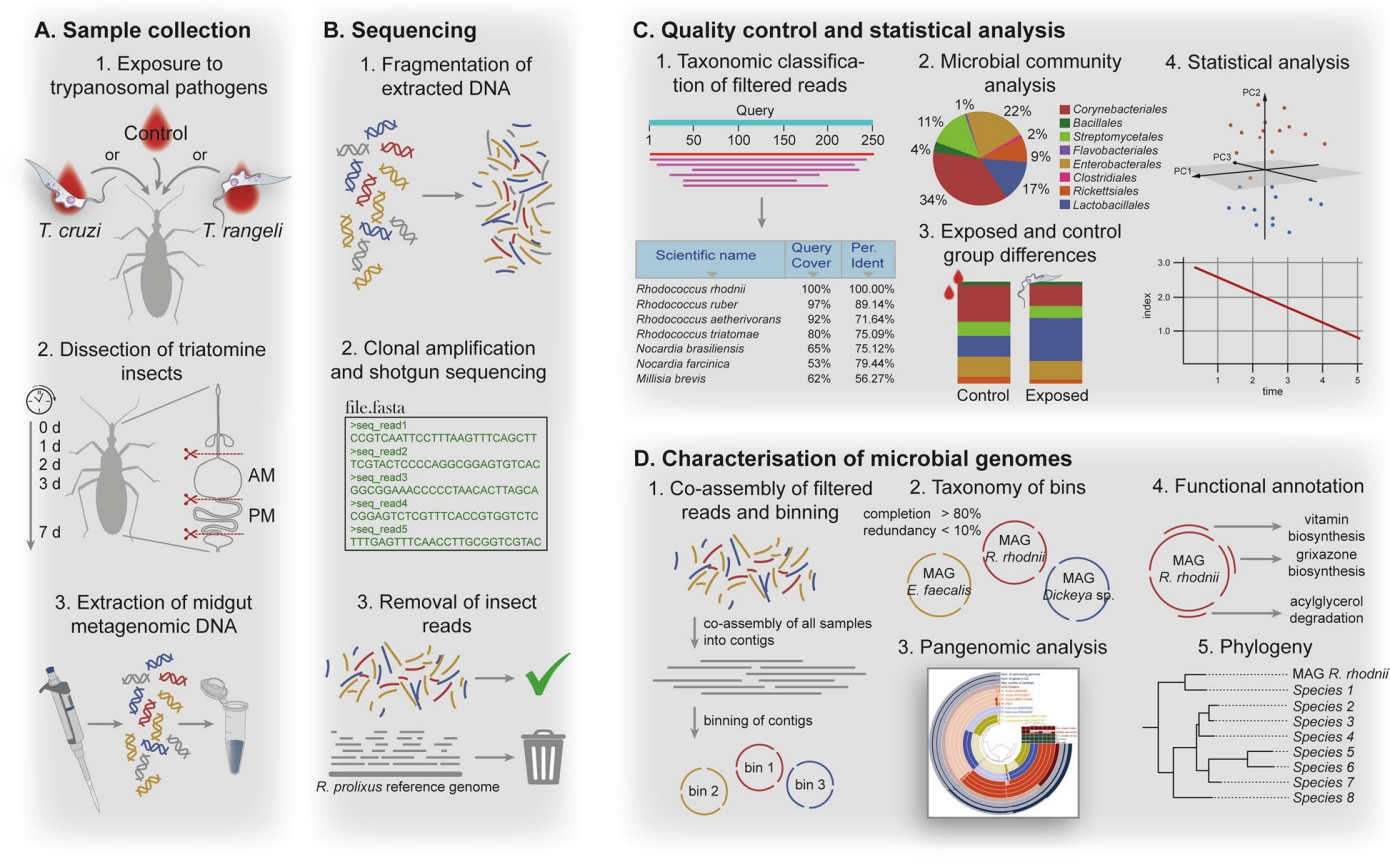
Applied methodology for data analysis. **A** Preparation and collection of the metagenomic DNA samples of the midgut of *R. prolixus*. **B** Shotgun sequencing and filtering of the reads. **C** Quality check and statistical analysis of the microbial community. **D** In-depth analysis of reconstructed MAGs

*Trypanosoma* infection was performed using *T. cruzi* strain Dm28c originally isolated from naturally infected opossum [29] and *T. rangeli* CHOACHI strain isolated from naturally infected *R. prolixus* [30]. Parasites were cultured in vitro by two weekly passages in liver-infusion tryptose (LIT) medium supplemented with 15% foetal bovine serum (FBS), 100 mg/ml streptomycin and 100 units/ml penicillin. In order to prevent loss of infectivity, parasites were submitted to cycles of triatomine-mice infection every six (*T. cruzi*) and three (*T. rangeli*) months [31]. For *T. cruzi* infection, SWR/J mice were intraperitoneally inoculated with 200 μl of triatomine urine containing ~5 × 10^4^ metacyclic trypomastigotes/ml. Parasitemia in the mice was 7–15 trypomastigotes/μl. For *T. rangeli* infection, SWR/J mice were anaesthetised with ketamine (150 mg/kg; Cristália, Brazil) and xylazine (10 mg/kg; Bayer, Brazil) and exposed to the bite of 5th instar nymphs containing trypomastigote forms in their salivary glands. Mice parasitemia was ~2.5 × 10^3^ trypomastigotes/ml. At day 9 (*T. cruzi*) and 14 (*T. rangeli*) after exposure, mice were anaesthetised and exposed to 5th instar nymphs of *R. prolixus* (30 days starved) until fully engorged. The same procedure was used for the control group with a healthy mouse (Fig. 1A-1).

**Fig. 2:**
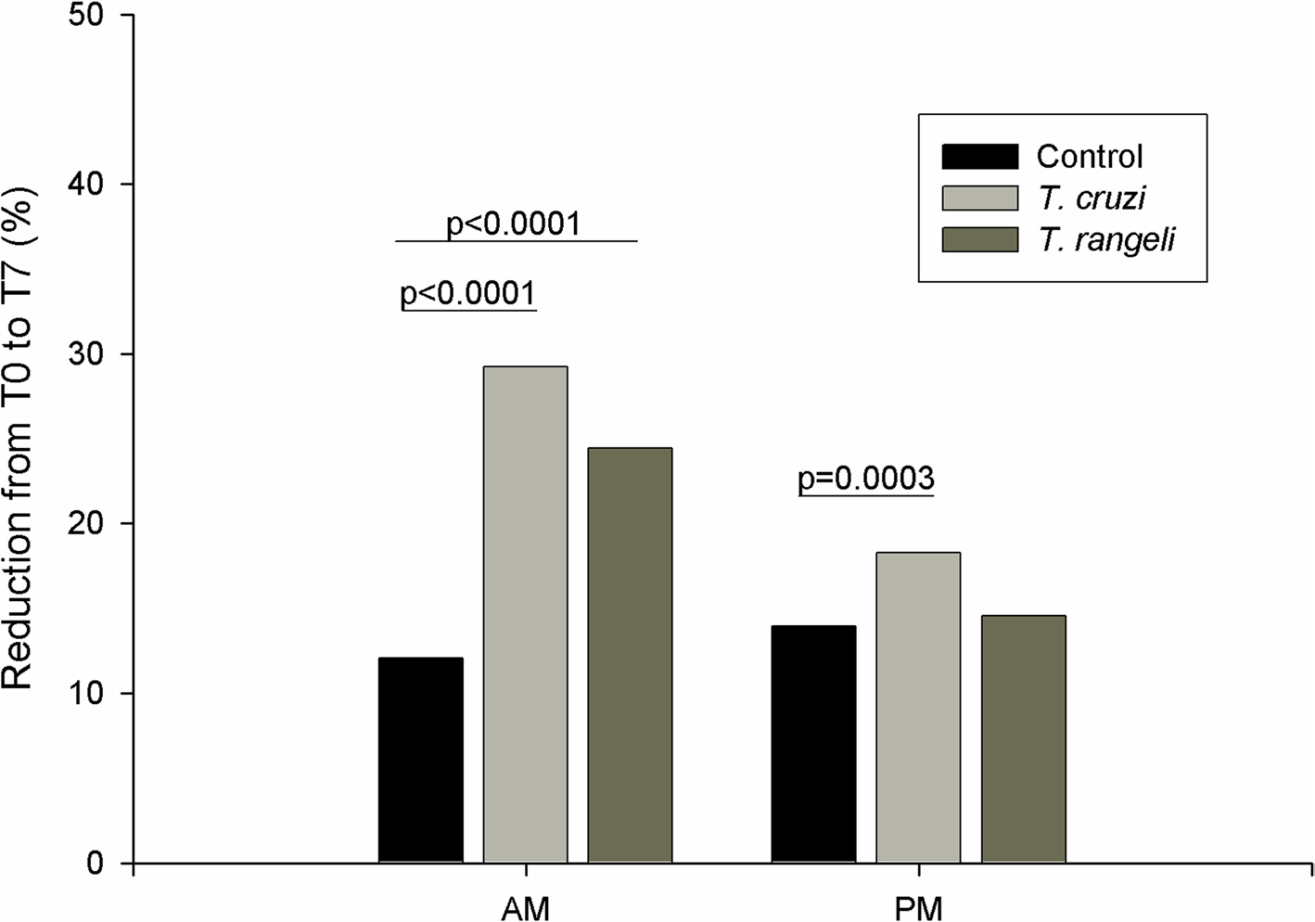
Reduction in the abundance of identified organisms in the anterior (AM) and posterior (PM) midgut of *T. cruzi*- and *T. rangeli*-exposed insects in comparison with nonexposed insects from T0 to T7 after exposure

Triatomine gut dissection followed immediately (timepoint 0, T0) as well as 1 day (24 h, timepoint 1, T1), 2 days (48 h, timepoint 2, T2), 3 days (72 h, timepoint 3, T3) and 7 days (168 h, timepoint 7, T7) after the blood meal and was performed using a dissecting microscope (Motic, SMZ-168) and sterile instruments. The intestinal compartments were segregated into anterior midgut (AM) and posterior midgut (PM) and pooled in samples with three individuals to yield a sufficient amount of intestinal DNA for metagenomic sequencing (Fig. 1A-2). In total, 45 insects were used resulting in 15 samples of the AM and 15 samples of the PM.

### DNA extraction and sequencing

**Fig. 3:**
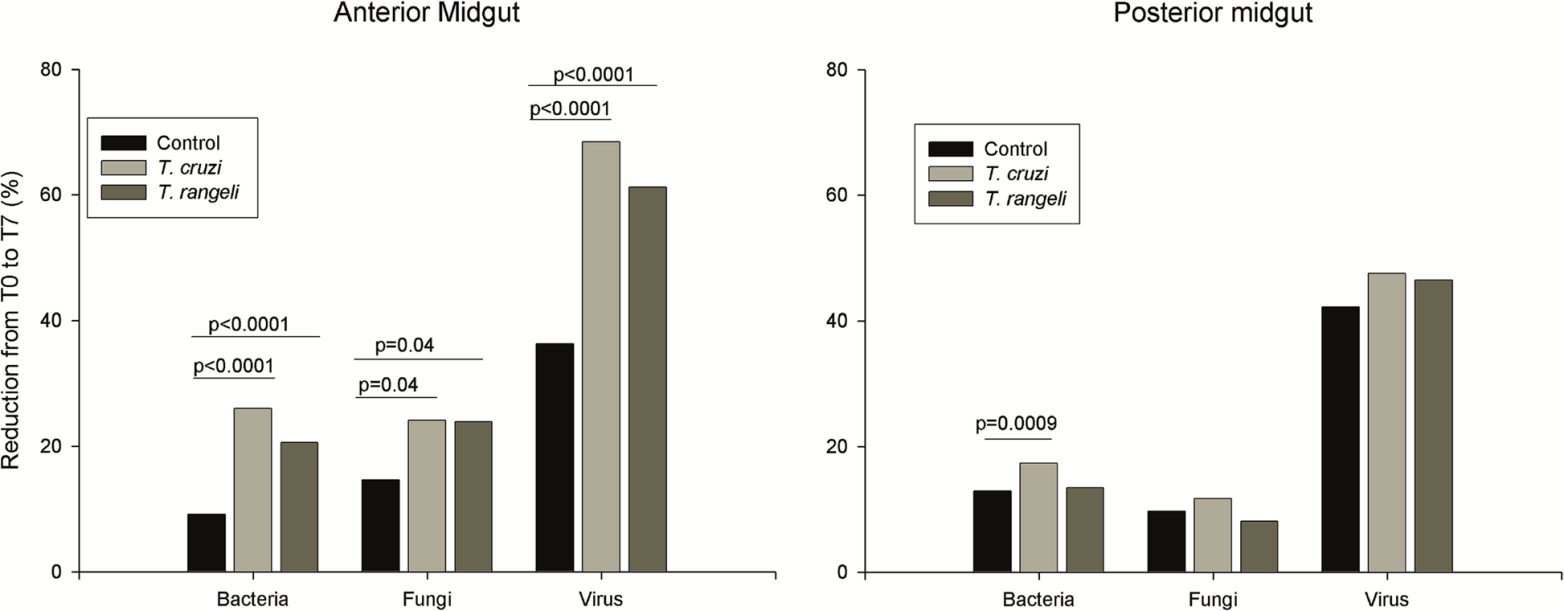
Reduction in the abundance of identified bacteria, fungi and viruses in the anterior (AM) and posterior (PM) midgut of *T. cruzi*- and *T. rangeli*-exposed insects in comparison with nonexposed insects from T0 to T7 after exposure

DNA of all intestinal samples was extracted using the AllPrep DNA/RNA Mini Kit (Qiagen) according to the manufacturer’s recommendations (Fig. 1A-3). The purified DNA was shipped to Novogene, and 30 libraries, one for each sample, were prepared by Novogene. Preparation of the samples was carried out using the NEB Ultra ll DNA Library preparation kit with four amplification cycles. Shotgun metagenomic sequencing was performed on an Illumina NovaSeq 6000 instrument for paired-end (150 bp, 300 cycles) reads (Fig. 1B-1 and B-2).

### Sequence processing and data analysis

**Fig. 4:**
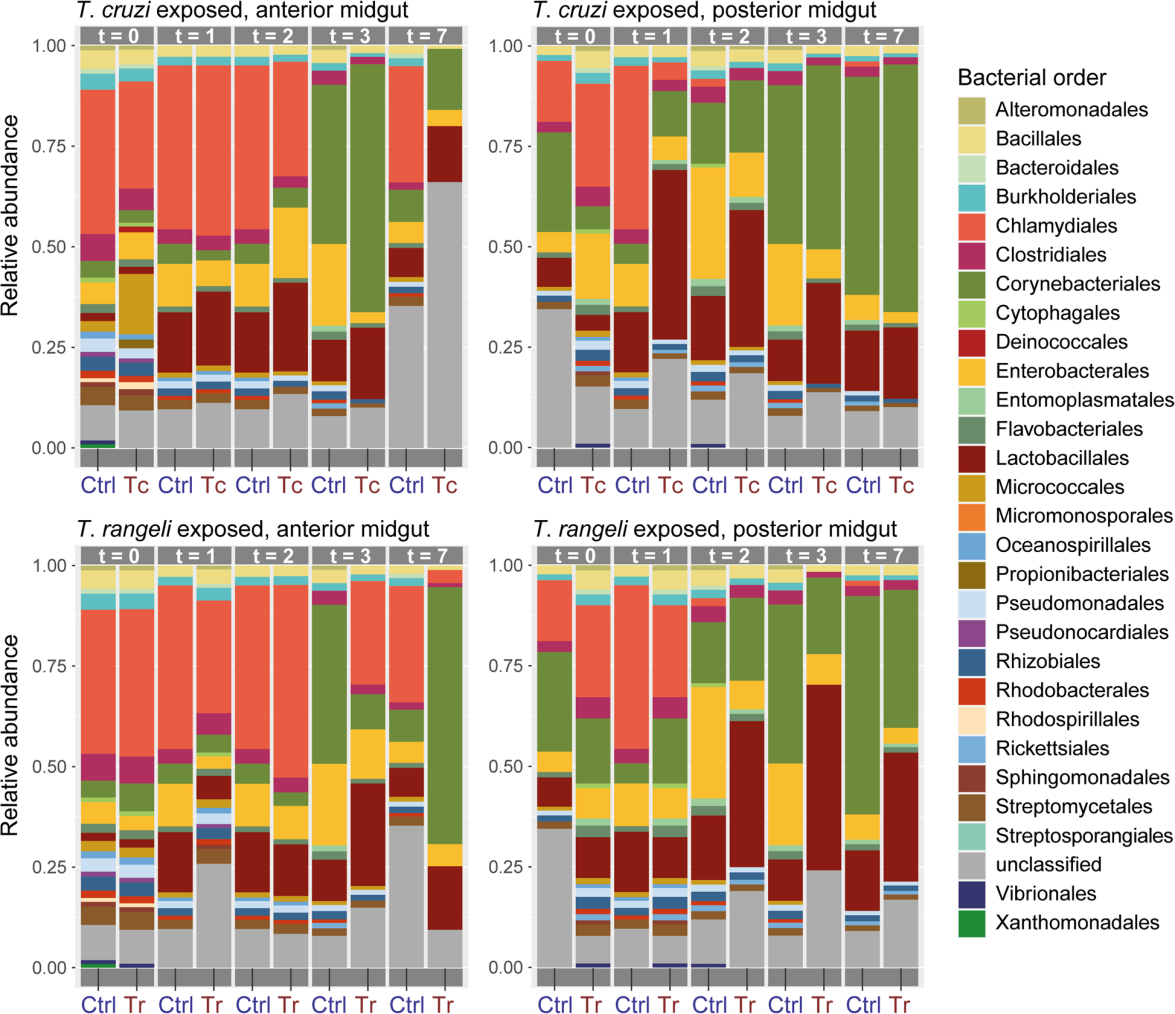
Relative abundance of bacterial orders in *T. cruzi*-exposed (Tc), *T. rangeli*-exposed (Tr) and nonexposed (Ctrl) anterior and posterior midgut samples of *R. prolixus* at days 0, 1, 2, 3 and 7 after exposure. The figure was created with the package *ggplot2* (v.3.3.3) in R

Quality control of the obtained raw-reads was implemented by using Trimmomatic (v.0.36) [32] removing low-quality bases and reads lacking a pair. Options were set to ILLUMINACLIP:TruSeq3-PE.fa:2:30:10 to remove adapters, LEADING 3, TRAILING 3 and SLIDINGWINDOW:4:15 to cut reads when average quality per base drops below 15 and MINLEN:36 to remove reads shorter than 36 bases. Taxonomic classification of the trimmed reads was achieved by using Kaiju web server [33] filtering against the nonredundant protein database of NCBI including fungi and microbial eukaryotes (Fig. 1C-1). The run mode was set for maximum exact matches with a minimum match length of 11. Results were processed in R (v.4.0.3) [34] and visualised using the *ggplot2* package (v.3.3.3) (Fig. 1C-2) [35]. A threshold was set which removed taxonomic hits with less than 0.7% of the assigned reads. Principle component analysis (PCA) was performed with the help of the R packages *data.table* (v.1.13.6) [36], *tidyverse* (v.1.3.0) [37] and *pca3d* (v.0.10.2) [38], using the relative abundance of bacterial orders to reduce data dimensionality while retaining as much of the compositional variance among samples as possible. The abundance of organisms from T0 to T7 was compared by using a Pearson’s chi-squared test. In order to assess bacterial alpha diversity and evenness, Shannon index and Pielou index were calculated (R package *vegan* v.2.5-7) (Fig. 1C-4) [39]. An unpaired *t-*test was used to determine the differences in the relative abundance of *Enterococcus* between the control group and *T. cruzi*- and *T. rangeli*-exposed insects as this genus was the most abundant taxon of *Lactobacillales* (Fig. 1C-3). Beforehand, the data was tested for normal distribution using the Shapiro-Wilk test and for homogeneity of variances using the Bartlett’s test (R package *stats* v.4.0.3). The trimmed reads were also mapped against the *R. prolixus* genome (GenBank assembly accession: GCA_000181055.3) collecting reads not mapping to the reference (option — un-conc) using bowtie2 (v.2.2.5) [40]. These reads were considered to be noninsect reads and were taken into account for further microbial community analysis (Fig. 1B-3). A co-assembly of all 30 samples was created using MEGAHIT (v.1.1.4) [41] with the −kmin-1 pass option activated (Fig. 1D). In order to retain sample specific information of contigs such as relative abundance of sequences and mean coverage, BAM files were generated by mapping reads from individual samples back to the co-assembly using bowtie2 (v.2.2.5) and SAMtools (v.1.2) [42].

The co-assembly was then used as an input for the Anvi’o (v.7) [43] metagenomic pipeline creating a contigs database and identifying open reading frames using Prodigal (v.2.6.3) [44]. Furthermore, bacterial single-copy core genes (SCG) were detected by running hidden Markov models (anvi-run-hmms), while genes were functionally annotated by DIAMOND (v.2.0.6) [45] using NCBI clusters of orthologous groups (COGs). Implemented in the anvi-estimate-scg-taxonomy command, DIAMOND was also used to rapidly determine taxonomy based on SCGs from the Genome Taxonomy Database (GTDB) [46]. Taxonomic elucidation was backed by centrifuge (v.1.0.4) [47] and added to the contigs database using the script anvi-import-taxonomy-for-genes (Fig. 1D-2). Sorted and indexed BAM files were profiled (anvi-profile) removing contigs shorter than 2500 nucleotides and recovering single-nucleotide variants and coverage information for each contig. Profile databases for every sample were merged into a single database with anvi-merge. Finally, the metagenomic contigs were categorised by the unsupervised automated binning tool CONCOCT (v.1.1.0) [48] utilising nucleotide composition, coverage data and linkage data from paired end reads. The resulting bin collection was imported into the profile database and manually inspected and refined based on predicted taxonomy, including blastx searches, read coverage and GC-content of contigs. Bins were removed from the collection when taxonomic results indicated mouse, chicken or other mammal sources derived from previous blood intakes.

### Pangenomic analysis

**Fig. 5:**
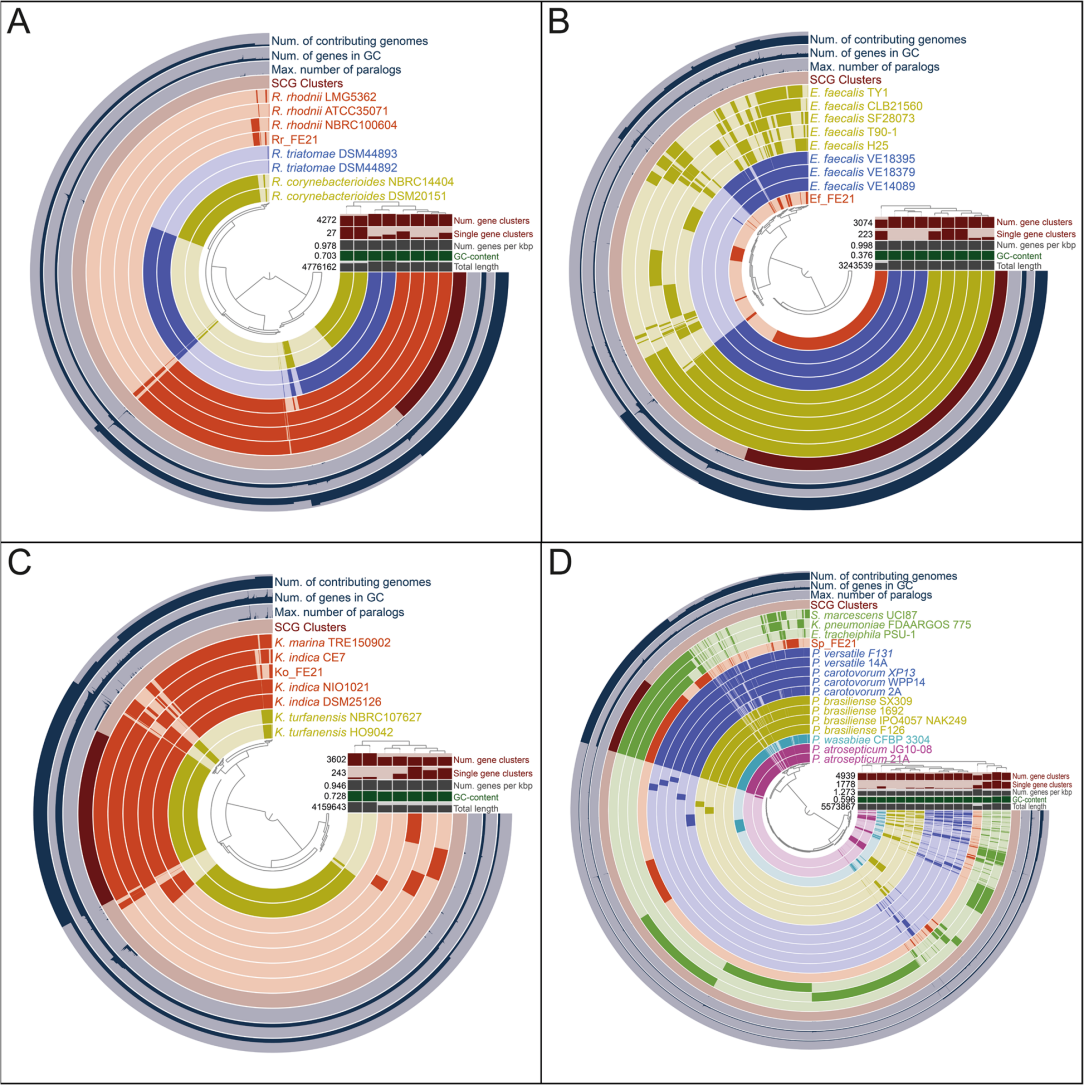
Pangenomic analyses of the metagenome-assembled genomes and closely related bacterial species obtained from NCBI. **A** Rr_FE21. **B** Ef_FE21. **C** Ko_FE21. **D** Sp_FE21. The colour-coded layers represent the gene clusters of the indicated species. The number of contributing genomes, the number of genes in the respective gene cluster, the maximum number of paralogs and the presence of SCG clusters are also given for each gene cluster. The columns indicate the number of gene clusters for each genome, the single gene clusters, the number of genes per 1000 bp, the GC-content and the total length of the genomes. The centrally located tree shows each split of the gene clusters, while the tree on the right is based on the gene cluster frequencies in each genome. Accession numbers of reference genomes and bacterial strains used are provided in Additional file 1

Assembled genomes of different *Enterococcus faecalis* strains, *Rhodococcus* species, *Kocuria* species and *Enterobacteriaceae* species were downloaded from NCBI [49] and converted into contigs databases including SCG identification and annotation of NCBI COGs (see Additional file 1 for bacterial strains used and accession numbers). Genomes were selected for analysis based on previous SCG hits by DIAMOND and their ecological and economical significance. For example, *Pectobacterium* was particularly chosen for the pangenome analysis of Sp_FE21 as single-copy core genes suggested a taxonomic coherence and several *Pectobacterium* species show high economic importance as plant pathogens. Pangenomic analyses of metagenome-assembled genomes (MAG) were performed using the Anvi’o pangenomic workflow [50–52] with the following options: −use-ncbi-blast activated, −mcl-inflation 8 (for pangenomic analysis on species level) and −mcl-inflation 10 (for pangenomic analysis on strain level) to adjust the algorithm’s sensitivity in detecting gene clusters and −minbit 0.5 to eliminate weak matches between two amino acid sequences (Fig. 1D-3) [53]. Gene clusters unique to Sp_FE21 were blasted against the nonredundant protein database of NCBI (blastp). A phylogenomic tree of Sp_FE21 was created using anvi-get-sequences-for-hmm-hits to extract nucleotide sequences of 71 SCG from Sp_FE21 and other soft rot causing *Enterobacteriaceae*. A sequence alignment of homologs was built with MAFFT 7 (v.7.490) and the activated option “scoring matrix for nucleotide sequences: 1 PAM/κ = 2” for closely related species [54]. Phylogeny was reconstructed by using a maximum likelihood approach and the “general time-reversible” model with invariant sites with MEGA7 (v.7.0) and tested by bootstrapping with 1000 replications [55] (Fig. 1D-5) (see Additional file 2 for genomes used and accession numbers).

### Functional analysis

In addition to the functional annotation with NCBI COGs, the Kyoto Encyclopedia of Genes and Genomes (KEGG) [56–58] was also used to reveal the metabolic capacities present in the assembled genomes from the samples (Fig. 1D-4). For this purpose, HMM profiles were downloaded from the database of KEGG orthologs (KOfam) [59] as well as metabolic information stored in the KEGG module database. Gene calls were extracted from the contigs database and tested for HMM hits (anvi-run-kegg-kofams). The completeness of the metabolic pathways (KEGG modules) was then reconstructed by the presence of the associated gene functions (anvi-estimate-metabolism), whereby a module was considered complete when 75% of its gene functions were detected. Subsequently, the output was used to create a heatmap of the metabolic capacities in R using the packages *RColorBrewer* (v.1.1.2) [60], *tidytable* (v.0.5.8) [61] and *pheatmap* (v.1.0.12) [62].

### Data availability

Sequencing data for all samples is available at NCBI Sequence Read Archive (SRA) under the BioProject accession PRJNA744378 with the individual identifiers provided in Additional file 3. Original R scripts used for downstream analysis can be obtained from GitHub [63]. Metagenome-assembled genomes have been deposited in NCBI BioSample and are available under the accessions SAMN20089395, SAMN20089396, SAMN20089397 and SAMN20089398.

## Results

With the aim of studying the effects of the protozoan parasites *T. cruzi* and *T. rangeli* on the microbiome of triatomine vectors, 30 pools, each with either three anterior or three posterior midguts from laboratory-reared *R. prolixus*, were prepared. One-third of the insects was challenged with *T. cruzi*, a third with *T. rangeli* and another third served as a nonexposed control group. The infection of the insects was observed over a period of 1 week, and samples were used for metagenomic shotgun sequencing on timepoints 0,1, 2, 3 and 7 after exposure resulting in an average of ~16 million read pairs per sample after trimming and the removal of insect reads (Additional file 4). Assembling the reads of all samples into one de novo co-assembly yielded 538,565 contigs larger than 1000 bp (max 467,137; N_50_ 11,671). For annotation, only contigs larger than 2500 were kept and binned into 56 genomic bins by CONCOCT and after manual refinement. Prodigal identified a total of 2,134,935 gene calls of which 21,045 were provided with a unique functional call by DIAMOND.

### Microbial community and pathogen exposure

The overall microbial diversity of the gut samples was assessed by running Kaiju web server on the trimmed reads. On average, 30.51% (SD 9.82%) of the reads of each sample were assigned to a taxonomic group (Additional file 5). Comparing the exposed samples and the control group, a reduction of 12% and 14.9% in the abundance of identified organisms in the AM and PM, respectively, was observed in the control group from T0 to T7. This reduction was significantly increased in the AM of *T. cruzi*- and *T. rangeli*-exposed insects (Fig. 2; *T. cruzi*-exposed *X*^2^ (1, *N* = 92565) = 265.95, *p* < 0.0001; *T. rangeli*-exposed *X*^2^ (1, *N* = 96089) = 136.41, *p* < 0.0001). In the PM, an increased reduction was only observed in *T. cruzi*-exposed insects (Fig. 2; *X*^2^ (1, *N* = 75828) = 12.74, *p* = 0.0003). When the numbers of identified bacteria, fungi and viruses were evaluated separately, a significant reduction from T0 to T7 was observed for all of them in the AM of both *T. cruzi*- and *T. rangeli*-exposed insects (Fig. 3; *T. cruzi*-exposed bacteria *X*^2^ (1, *N* = 76752) = 198.72, *p* < 0.0001; fungi *X*^2^ (1, *N* = 4870) = 4.10, *p* < 0.04; viruses *X*^2^ (1, *N* = 5358) = 128.56, *p* < 0.0001; *T. rangeli*-exposed bacteria *X*^2^ (1, *N* = 79279) = 88.97, *p* < 0.0001; fungi *X*^2^ (1, *N* = 5020) = 4.09, *p* < 0.04; viruses *X*^2^ (1, *N* = 5910) = 78.52, *p* < 0.0001). In the PM, a significant reduction in the number of bacteria was observed in *T. cruzi*-exposed insects (Fig. 3; *X*^2^ (1, *N* = 64677) = 10.96, *p* = 0009).

Approximately, 73.87% (SD 20.74%) of the assigned reads had a bacterial origin, from which 28 different bacterial orders from twelve classes were detected (Fig. 4). The most abundant classes of bacteria were *Chlamydiae*, *Actinobacteria*, *Firmicutes* and *Gammaproteobacteria* in descending order. The samples of the AM were dominated by *Chlamydiales*, in particular at the first three timepoints (days 0, 1 and 2 after exposure). Afterwards, the distribution partially shifts towards *Corynebacteriales*, *Enterobacterales* and *Lactobacillales*. The *Chlamydiales* have a considerably lower ratio in the PM, whereas the aforementioned orders occur more frequently and at earlier timepoints leading to a notable separation between AM and PM sample composition revealed by PCA (Additional file 6A). At later timepoints, *Corynebacteriales* and *Lactobacillales* appear as the very predominant orders in the samples of the AM as well as the PM. Accordingly, the Shannon index shows a lower alpha diversity of bacterial orders of the pooled insect samples at later timepoints, while Pielou’s index also indicates a lower species evenness of the pooled samples. Interestingly, this difference is much more pronounced in the exposed samples than in the nonexposed samples (Additional files 6B and 7).

Further differences between exposed and control group are evident in the bacterial species composition. In the PM samples at T1, unlike the control group, which is mainly composed of *Enterobacterales*, *Chlamydiales*, *Clostridiales*, *Corynebacteriales* and *Lactobacillales*, the sample challenged with *T. cruzi* is largely dominated by *Lactobacillales*. This is also the case at T2 and T3 with decreasing distinctness. Finally, the bacterial composition between exposed and control group at T7 is again resembling, with *Corynebacteriales* as the predominant order. These changes are more pronounced in case of the PM samples challenged with *T. rangeli*, even at later timepoints where the *Lactobacillales* are clearly the predominant bacterial order (Fig. 4). A closer look into the detected *Lactobacillales* shows that they are mostly represented by the genus *Enterococcus* in both *T. cruzi*- and *T. rangeli*-exposed samples. An unpaired *t*-test was conducted to compare the relative abundance of reads assigned to *Enterococcus* between the control group and the exposed groups at T2 to T7. The results showed a statistically significant difference suggesting a higher relative abundance of *Enterococcus* in both *T. cruzi*-exposed insects (t(10) = −2.9403, *p-*value < 0.05) and *T. rangeli*-exposed insects (t(10) = −2.7497, *p-*value < 0.05) (median ± SD, control group 4.75 ± 1.36; *T. cruzi*-exposed 7.87 ± 2.22; *T. rangeli*-exposed 8.98 ± 3.51).

### Metagenome-assembled genomes and pangenomic analysis

In total, four MAGs were recovered from the genomic bins of which three, Ef_FE21, Rr_FE21 and Ko_FE21, were rapidly classified as *Enterococcus faecalis*, *Rhodococcus rhodnii* and *Kocuria* spp. based on SCG annotation by DIAMOND [45], respectively. The fourth MAG could not be clearly assigned to a species, but according to SCGs, it belonged to the *Enterobacteriaceae* family. Completion and redundancy values were estimated, reporting bins featuring > 80% completion and < 10% redundancy as a MAG. Ef_FE21, with a total length of 2.44 Mb and GC-content of 37.01%, was calculated to be 97.2% complete and 2.8% redundant. Rr_FE21 showed 100% completion and 1.4% redundancy with a total length of 4.3 Mb and GC-content of 69.27%. Both were primarily found in the PM at later timepoints after exposure. The completion and redundancy values for Ko_FE21 (length, 2.86 Mb; GC-content, 69.16%) were 98.6% and 0%, respectively, while Sp_FE21 (length, 3.29 Mb; GC-content, 50.31%) was calculated to be 85.9% complete and 0% redundant. Ko_FE21 appeared only in the AM sample immediately after exposure with a very high relative abundance. Interestingly, as an exception, neither Ef_FE21 nor Rr_FE21 is substantially represented in this sample. Furthermore, the genomes of *Dickeya zeae* (completion 14.08%, redundancy 0%, genome length 221 Kb) and the common insect symbiont *Wolbachia* spp. (completion 2.82%, redundancy 0%, genome length 401 Kb) were only partially obtained.

**Fig. 6:**
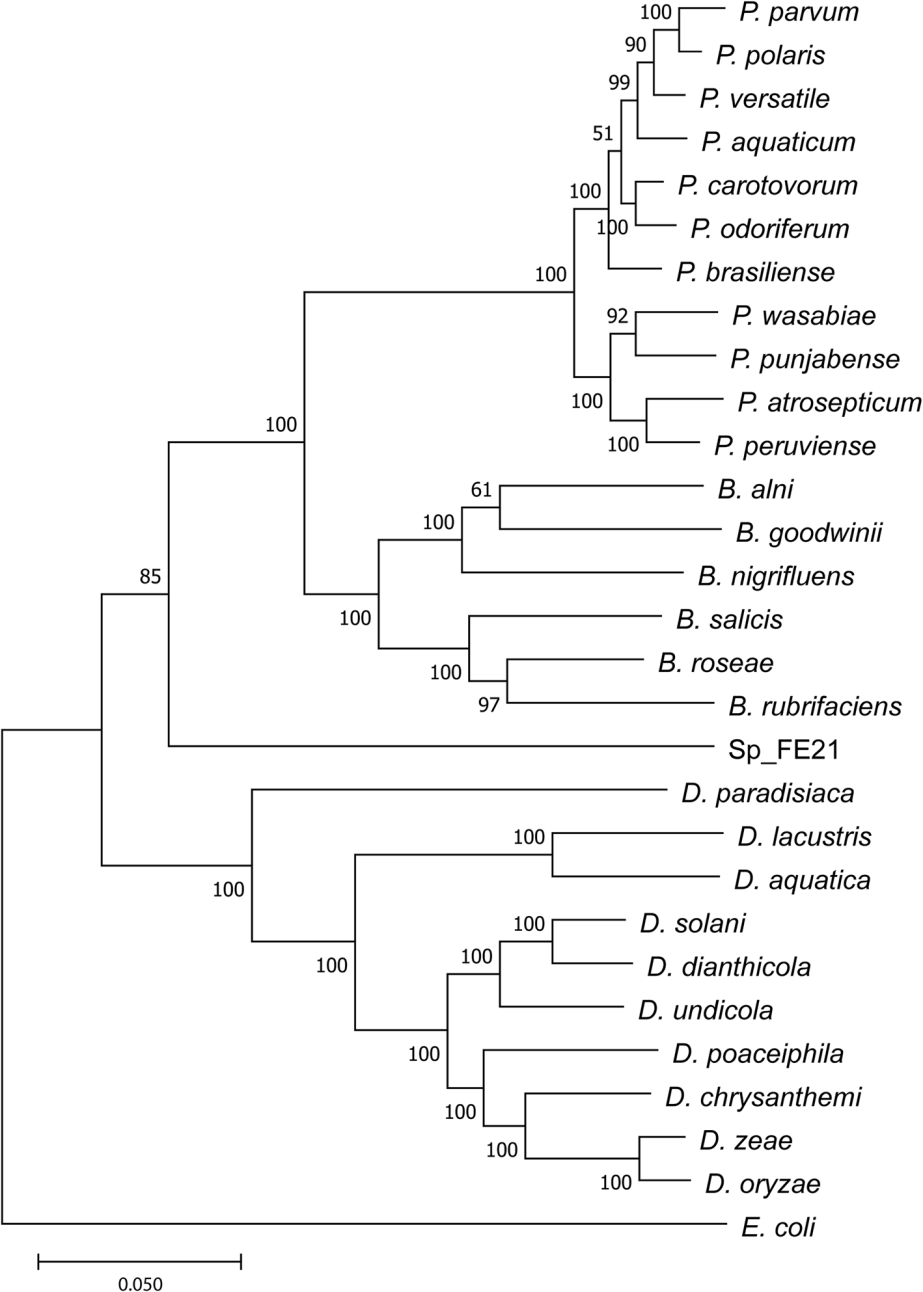
Phylogenomic tree of soft rot causing *Enterobacteriaceae* and Sp_FE21 based on homologous SCGs. Sp_FE21 presents as sister to the genera *Brenneria* and *Pectobacterium*. *Escherichia coli* was used as an outgroup in order to root the tree. Phylogeny was constructed using anvi-get-sequences-for-hmm-hits, MAFFT 7 and MEGA7 and tested by bootstrapping with 1000 replications. Confidence values are indicated

Pangenomic analysis of the different MAGs including assembled genomes of related species from NCBI supported the in-depth taxonomic classification and yielded information on genome length and both common and singleton gene clusters. As expected, based on gene cluster frequencies, Rr_FE21 was assigned to the *R. rhodnii* branch of the tree. It also shows a similar total length compared with the other used *R. rhodnii* genomes and has most of the common gene clusters leading to the assumption that the newly assembled genome is almost complete (Fig. 5A). Ef_FE21 could not be assigned to a specific *E. faecalis* strain. However, it turned out that it has less gene clusters than the reference genomes and is also shorter (Fig. 5B). Hence, we reason that the genome is unlikely to be complete. The pangenomic analysis of Ko_FE21 allocated the metagenome-assembled genome together with *Kocuria indica* and *K. marina*, which have a similar total length and number of gene clusters. A more precise classification was not achieved as *K. indica* and *K. marina* display various similarities on the gene cluster level (Fig. 5C). It should be noted, however, that the taxonomy check of *K. marina* by NCBI was classified as inconclusive. The pangenomic analysis of Sp_FE21 confirmed that it is most likely part of the *Enterobacteriaceae* family; nevertheless, a specific genus was not revealed. It was not found to match to any of the *Pectobacterium* species used, nor to the added genomes of other widespread *Enterobacteriaceae* such as *Serratia marcescens*, *Klebsiella pneumonia* or *Erwinia tracheiphila*. Furthermore, the length as well as the number of gene clusters present suggests that Sp_FE21 is approximately 50% complete (Fig. 5D). Aligning the extracted amino acid sequences of gene clusters unique to Sp_FE21 with nonredundant protein databases (blastp) indicated a close genetic congruence with the newly described insect symbiont *Candidatus Symbiopectobacterium*. Phylogenomic analysis based on bacterial SCG of different soft rots causing *Enterobacteriaceae* suggests that Sp_FE21 belongs to a sister clade of the genera *Brenneria* and *Pectobacterium* (Fig. 6).

### Metabolic capacity of the vector’s microbiome

**Fig. 7:**
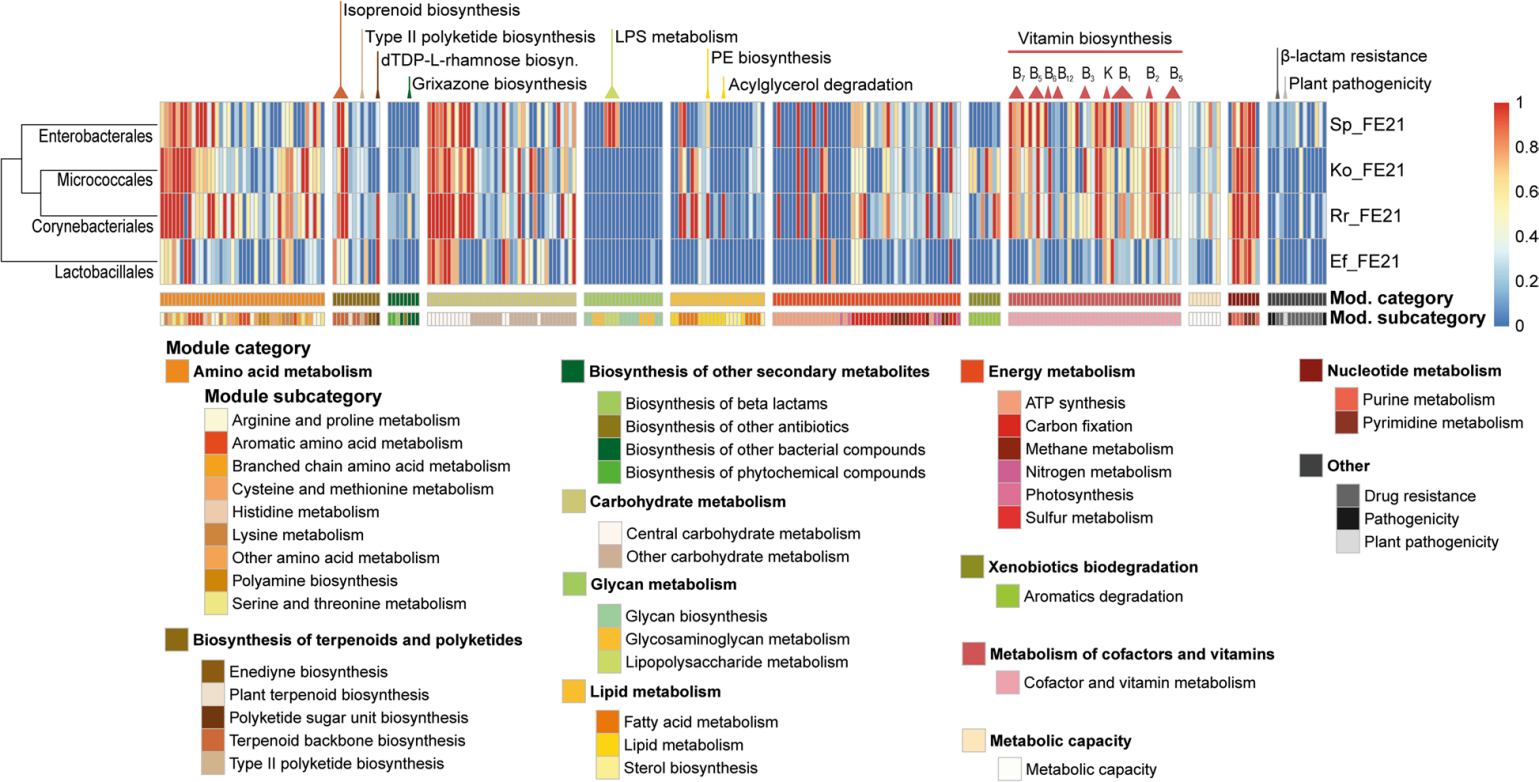
Comparison of the completeness of functional pathways present in the MAGs Re_FE21, Ef_FE21, Ko_FE21 and Sp_FE21. KEGG module categories and subcategories are shown with remarkable pathways highlighted. The figure was created with the package *pheatmap* (v.1.0.12) in R

The functional pathways (KEGG modules) present in the obtained MAGs were reconstructed based on the completion of gene function sets from KEGG (Additional file 8). A total of twelve module categories were considered, each of which was split into subcategories providing insight into the diverse functional capabilities of each assembled genome. It was not surprising that fundamental constitutive gene sets such as different pathways of the amino acid metabolism, carbohydrate metabolism, energy metabolism and nucleotide metabolism were similarly distributed in all MAGs (Fig. 7). However, even within basic metabolic modules, differences in the presence of submodules were detected. For instance, only Rr_FE21 possesses a complete pathway for the degradation of acylglycerol as part of the lipid metabolism. The same is evident regarding the biosynthesis of phosphatidylethanolamine (PE) from phosphatidic acid (PA). Lipopolysaccharide (LPS) biosynthesis is solely present in Sp_FE21 indicating a gram-negative bacterium such as members of the *Enterobacteriaceae* family. The presence of a complete module associated with plant pathogenicity, as it might occur in the plant soft rot causing bacterium *Pectobacterium*, has not been determined. In all MAGs, but especially in Ko_FE21, Rr_FE21 and Sp_FE21, there is a wide variety of gene sets involved in the metabolism of vitamins and cofactors. These include enzymes that are responsible for the biosynthesis of thiamine (vitamin B_1_), riboflavin (vitamin B_2_), niacin (vitamin B_3_) and coenzyme NAD, pantothenate (vitamin B_5_) and coenzyme A, pyridoxal (vitamin B_6_), biotin (vitamin B_7_), the cofactor tetrahydrofolate (folate/vitamin B_9_ derivative), cobalamins (vitamin B_12_) and vitamin K_2_. Furthermore, complete and partially complete gene modules of secondary metabolites, which code for the biosynthesis of terpenoids and polyketides as well as other bacterial compounds, were elucidated. These natural products are not directly involved in the development or reproduction of an organism but are produced to gain a selective advantage and display broad biological activities [64]. They are often restricted to a taxonomic group; thus Ko_FE21 is the only MAG investigated to have genetic traits for the biosynthesis of type II polyketides (PKs) covering a third of the gene set including an ortholog for *act*IV cyclase (aldolase). The gene cluster of the precursor of aminoglycoside streptomycin and cytotoxic enediyne antibiotics, dTDP-L-rhamnose, is present in Rr_FE21 and Ef_FE21 as part of the polyketide sugar unit biosynthesis. Besides, Ef_FE21 also owns an incomplete gene cluster for the resistance against β-lactam antibiotics. Terpenoid backbone production is supported by all considered MAGs regarding C5 isoprenoid and C10-C20 isoprenoid biosynthesis. Additionally, a partially complete gene cluster for the production of the phenoxazine grixazone B was found in Rr_FE21.

## Discussion

In recent years, the advances of sequencing technology have found their way into research on microbial communities of vector-transmitted human pathogens and their associated hosts. Previously, 16S rRNA gene amplicon sequencing was the method of choice, but approaches which target entire metagenomes are now increasingly being deployed [65–68]. We used metagenomic shotgun sequencing to study the alterations of the intestinal microbiome of the Latin American vector, *R. prolixus*, induced by the exposure to *T*. *cruzi* and *T. rangeli*. Consistent with other studies on insect microbiota using metagenomic shotgun sequencing, our biocomputational analysis identified high abundances of bacterial reads far exceeding those of fungal, protozoan and viral origin [69–72]. This might be due to a bias in the reference database used, in which life forms other than bacteria are underrepresented, as shown by LaPierre et al. [73].

In addition to *Bacilli* and *Bacteroidetes*, the bacterial classes *Actinobacteria*, *Firmicutes* and *Gammaproteobacteria* have frequently been detected as part of the microbial community of triatomine insects, often with *Actinobacteria* as the most abundant class [7, 72, 74–76]. Here, the *Actinobacteria* are mainly represented by *Corynebacteriales*, the *Firmicutes* are mostly comprised of *Lactobacillales* and the *Proteobacteria* are largely constituted of *Enterobacterales*. Most have been previously shown to be members of the triatomine microbiota, albeit not in *R. prolixus* [77–79]. According to current 16S rRNA gene amplicon studies, the microbiota of *R. prolixus* is mostly dominated by *Pectobacterium* (*Enterobacterales*) and *Rhodococcus* (*Corynebacteriales*), while the comparison of the microbial diversity usually shows low intraindividual variations and high interindividual variations. Furthermore, *Staphylococcus* (*Bacillales*), *Serratia* (*Enterobacterales*) and *Wolbachia* (*Rickettsiales*) were identified as relevant representatives of the *R. prolixus* microbiota [74, 79, 80]. The obligate intracellular bacteria *Wolbachia* spp. are common in a wide range of insects, including sand flies, bed bugs, fleas and mosquitoes, and can cause reproduction alterations such as feminization, male killing and cytoplasmic incompatibility [81]. In triatomines, *Wolbachia* has been solely reported for the genus *Rhodnius*, where it occurs in the intestine, salivary glands and gonads [82, 83]. The samples of the AM at early timepoints after exposure are dominated by *Chlamydiales*, while the influence of this bacterial order decreases or does not exist at all in the PM and at later timepoints indicating a merely blood meal-derived origin. In fact, infections with the sexually transmitted *Chlamydia* bacteria are common in laboratory mice and mice kept for feeding [84].

A difference in the microbiota composition of triatomines on a taxonomic level between infected and noninfected insects has rarely been demonstrated before [85]. However, taking into account the overall bacterial diversity, Díaz et al. [79] showed that *T. cruzi*-challenged insects bare a more diverse bacterial community than the control group. In contrast, our results describe a lower number of organisms in general, and a decreasing alpha diversity and species evenness over the timescale of the study in both *T. cruzi* and *T. rangeli* exposed insects (Additional file 6B). This decline might be due to the progressing metabolic breakdown of the blood and the subsequent reduced supply of nutrients such as iron and proteins in the triatomine intestine inhibiting the proliferation of the bacterial community [25, 28]. Interestingly, both alpha diversity and species evenness decrease more strongly in the challenged insect’s samples compared to control samples. The same applies to the total number of organisms suggesting an influence of the exposure status on the overall microbial community. These effects might be related to the developmental cycle of the two pathogens. Despite belonging to same genus and sharing the same hosts, *T. cruzi* and *T. rangeli* differ in the way they develop in their hosts. In mammalian hosts, *T. cruzi* produces intracellular amastigote forms that multiply and differentiate into blood trypomastigotes, which will be transmitted to the triatomines during the blood meal [86]. The developmental cycle of *T. rangeli* in mammals is not yet known, and some evidence that multiplication occurs in secondary lymphoid organs has been published recently [87]. Differences are even more pronounced in invertebrate hosts. Once ingested, *T. cruzi* does not remain in the AM, which seems to be an inhospitable site for the parasite. When infecting *R. prolixus*, more than 80% of the parasites are killed in the AM within the first 24 h after ingestion [88, 89], while in infections in *T. infestans*, parasites rapidly cross the AM [90]. In both triatomine species, *T. cruzi* epimastigogenesis occurs in the PM, and no parasites are found in the AM after a few days of infection [88, 90]. In established infections, the development of the parasite occurs mainly in the rectum [91]. In the case of *T. rangeli*, the parasites differentiate into epimastigote forms in the AM, which is colonised, as well as the rest of the intestinal tract, in a few days after ingestion [92]. From day 0 to 7 after exposure, both parasites significantly decreased microbiome diversity in the AM of *R. prolixus* but possibly through different mechanisms. The infection of *R. prolixus* with *T. cruzi* DM28c strain triggers the activation of immune responses leading to an increase in the antimicrobial activity, levels of prophenoloxidase and AMP expression [25, 28, 93], which probably drives the reduction in the number of microorganisms found in the AM. In *T. rangeli* infections, the presence of the parasite in the intestinal tract promotes a systemic reduction of the activation of the Toll and IMD pathways, probably to allow the development of parasites that reach the hemocoel [94]. The production of the antimicrobial lysozymes A and B, and prolixicin, is also decreased in *T. rangeli*-infected *R. prolixus* [23]. Competition for nutrients or other unknown factors may be triggering the microbiome diversity reduction in this case, since massive populations of parasites can be found in the midgut of infected *R. prolixus* [92].

Shortly after the blood meal, especially in the AM, the most prevalent viruses detected were mammalian-related viruses, such as mammalian gammaretroviruses and mouse intracisternal A particle, which probably originated from the blood source. In all samples in the PM and also at later timepoints in the AM, a *Cotesia vestalis* bracovirus-like virus has been identified in high prevalence. This symbiotic virus, which has been detected in *R. prolixus* once, is closely associated with parasitic wasps and supports the exploitation of their lepidopteran host [95]. The most common fungi were different species of the genera *Aspergillus* and *Candida*, as well as the soil fungus *Mortierella* and the pathogenic *Coccidioides*. Especially shortly after the blood meal, the AM contains about three times the fungal diversity as the PM, which, however, balances out by timepoint 7.

The alterations of the bacterial microbiota at later timepoints are mainly driven by the relative abundance of *Corynebacteriales*, *Enterobacterales* and *Lactobacillales*, while the presence of *Corynebacteriales* and *Enterobacterales* clearly predominates in the control group, and the *Lactobacillales* are prevalent in the exposed group. These results are not atypical, as several members of the *Corynebacteriales* belong to the natural microbiota of triatomine insects, for example *Corynebacterium*, *Dietzia*, *Nocardia* and *Gordonia*, but also *R. rhodnii* [75, 76, 78, 96, 97]. The reconstructed MAG Rr_FE21 was classified as *R. rhodnii*, a common mutual symbiont of *R. prolixus*, which has been described as a member of its microbiome several times [74, 79, 98, 99]. Its extensive appearance in all samples is not surprising, since it has been postulated that the bacteria are important suppliers of vitamin B, a nutrient lacking in the natural diet of obligate blood-sucking insects [100]. Similar symbioses have already been revealed in a number of other haematophagous arthropods, such as bedbugs, tsetse flies and ticks [101–103]. The hypothesis is further supported by the fact that aposymbiotic triatomines show retardation of growth and fail to moult at late developmental stages, while the administration of symbiotic bacteria rectifies these effects [104–106]. However, the administration of auxotrophic *R. rhodnii* that is incapable of synthesising different B-group vitamins is sufficient to avoid a developmental delay indicating a subsidiary role of vitamin supplement by *R. rhodnii* [107]. In the present study, we were also able to assemble two other bacteria from the microbiome of *R. prolixus*, Ko_FE21 and Sp_FE21, which possess gene clusters for the production of a variety of B vitamins (Fig. 7). Similar results have been demonstrated for the genus *Dickeya* suggesting that *R. rhodnii* is unlikely to be the sole supplier of vitamin B-complex nutrients, as has long been assumed [104–106, 108]. In order to investigate the role of microbial organisms as symbiotic partners producing vital compounds for their insect hosts, it seems unavoidable to consider other metabolic feature groups than vitamins and cofactors [72, 109]. For instance, the degradation of acylglycerols as part of the lipid metabolism is solely found in the genome of Rr_FE21 suggesting that energy storage and mobilisation via acylglycerol are present in *R. rhodnii* as it was shown for other Rhodococci [110–112]. In this context, Vallejo et al. [109] also mentions mycolic acids, which are characteristic components of the cell wall and exclusively produced by actinomycetes. No fully assembled secondary metabolite gene cluster was found in Rr_FE21. We did, however, identify a partially assembled cluster reportedly responsible for the production of grixazone B, a yellow pigment that contains a phenoxazinone chromophore produced by the actinomycete *Streptomyces griseus* subsp. *griseus*. Surprisingly, the distribution of phenoxazinone synthases and their applications within the actinomycetes are relatively unexplored, with the industrial strain *R. jostii* RHA1, whose genome encodes for a putative phenoxazinone synthase, being the closest relative to Rr_FE21 with a comparable gene cluster [113, 114].

The significance of actinomycetes for the production of remarkable compounds is also highlighted by the MAG Ko_FE21, which only appeared in one sample with very high abundance, and was assigned to the genus *Kocuria*. Interestingly, compared to all others, this sample contained considerably less Rr_FE21 and Ef_FE21 associated reads raising the question if Ko_FE21 might outcompete regularly occurring bacterial symbionts in the intestinal tract of *R. prolixus*. The analysis of the functional capacities of Ko_FE21 revealed metabolic traits similar to those of Rr_FE21, especially regarding the biosynthesis of essential nutrients. However, the genus *Kocuria* also deserves attention due to the presence of secondary metabolite genes encoding for nonribosomal peptide synthetases (NRPSs) and polyketide synthases (PKSs) underlining their high competitiveness [115, 116]. This is emphasised by a gene cluster responsible for the biosynthesis of a type II polyketide found in Ko_FE21 (Fig. 7) and demonstrates the widespread existence of potent natural products throughout diverse environments, including insect hosts. However, since probably only a single lab-reared insect was colonised by Ko_FE21, it is inconclusive whether the bacteria are passed on within the colony. Pangenomic analysis failed to ascertain an exact species for Ko_FE21 but connected it to *K. indica* and *K. marina* (Fig. 5C). The actinomycete *K. indica* has been isolated from soil samples and human skin and has not been subject to detailed research yet, while the halophilic *K. marina* has been observed to cause inflammations in humans such as peritonitis and bloodstream infections and presents resistances against the antibiotics kanamycin, polymyxin B, benzylpenicillin and cotrimoxazole [117, 118]. This illustrates the potential for future research on groups of bacteria which have received less attention up to this point.

Representatives of the second predominant group in the control group, *Enterobacterales*, such as *Serratia*, *Arsenophonus* and *Pectobacterium* have also been shown to be members of the core triatomine microbiota [6, 74, 79, 119]. Hence, their presence in all intestinal samples of *R. prolixus* is not surprising at first. However, the in-depth pangenomic analysis of the MAG Sp_FE21, initially expected to be *Pectobacterium*, revealed a bacterial microbe which has not been detected in this form in triatomine microbiomes before. The presence of several gene clusters related to LPS metabolism identified it as a gram-negative bacteria. However, the analysis of the metabolic capacity of Sp_FE21 did not reveal genes encoding for plant pathogenicity as it would have been expected for the plant soft rot causing bacterium *Pectobacterium*. Furthermore, Sp_FE21 showed a similar GC-content compared to *Pectobacterium*, but its total genome length of 3.29 Mb was considerably shorter. Despite its small genome size, Sp_FE21 still had a large number of unique gene clusters which were assigned to *Candidatus Symbiopectobacterium* by protein database alignment. *Candidatus Symbiopectobacterium* has recently been described for the first time as an intracellular bacterial symbiont of the nematode *Howardula aoronymphium* parasitizing *Drosophila* flies. It was further found as an endosymbiont of other insects, such as mealybugs, leafhoppers and the bulrush bug (such as *Candidatus Rohrkolberia*) [120–123]. The genome of *Candidatus Symbiopectobacterium* shares large areas of gene order synteny with *P. carotovorum* indicating a sister clade of the plant-pathogenic genera *Pectobacterium*, *Dickeya* and *Brenneria*. Our phylogenomic analysis supports this conjecture by classifying Sp_FE21 in a monophyletic group with *Brenneria* and *Pectobacterium* (Fig. 6). Martinson et al. [122] described several disrupted amino acid and vitamin synthesis pathways in *Symbiopectobacterium* associated with the bulrush bug, whereas *Symbiopectobacterium* in nematodes has maintained most of its metabolic functions. Likewise, we were able to determine housekeeping genes and gene clusters connected to the biosynthesis of vitamins and cofactors in Sp_FE21 (Fig. 7). In previous studies on the microbiota of triatomine vectors, *Pectobacterium* as well as an unclassified *Enterobacteriaceae*, in rare cases referred to as *Candidatus Rohrkolberia*, were often found to be part of the core microbiome, particularly in the genus *Rhodnius*. However, most of these studies used amplicon sequencing of the hypervariable subregions V3/V4, V4/V5 and V6-V8 of the 16S rRNA gene, which can produce unreliable taxonomic classifications, especially within the *Enterobacteriaceae* [124, 125], and potentially led to a misguided identification of the endosymbiont, where in fact it might have actually been *Candidatus Symbiopectobacterium* [78, 79, 119, 126]. Accordingly, comparing and taxonomically classifying the different regions of the 16S rRNA gene of *Candidatus Symbiopectobacterium* and *Pectobacterium* resulted in high sequence similarities and a general erroneous identification as the genus *Pectobacterium* using SILVA [127, 128].

The order *Lactobacillales*, represented mostly by *E. faecalis* (Ef_FE21), has rarely been identified as part of the triatomine intestinal microbiota, and if so, it is typically a subordinate taxon. Its appearance as the most abundant order is therefore unusual, especially since it occurs increasingly in the pathogen-exposed samples. Accordingly, *E. faecalis* was previously detected only once as the predominant genus of the intestinal microbiota of lab-reared *Triatoma infestans* [6, 78, 108, 109, 129, 130]. Compared to wild-caught triatomines, these insects had a lower alpha diversity and were dominated by *E. faecalis*, an unclassified *Enterobacteriaceae* and *Bacillus* [78]. Normally, GC-rich bacterial genera outcompete GC-poor bacteria, including *Enterococcus*, in the digestive tract of triatomines due to their specific enzymatic abilities [126]. Our results, however, showed that the genus *Enterococcus* occurs more frequently in *R. prolixus* when it is exposed to trypanosomal pathogens, regardless of whether it is *T. cruzi* or *T. rangeli*, and even develop as the dominant taxonomic group. Therefore, *Enterococcus* may specifically multiply in exceptional situations when the natural balance of the triatomine microbiota cannot be established because of a reduced environment (laboratory) or a disturbance by pathogens. This is supported by the fact that different antibiotic resistances and virulence factors, such as vancomycin, streptomycin and β-lactam resistance, have been reported *for E. faecalis*, and that it occurs frequently as an opportunistic pathogen, especially in the nosocomial environment [131]. Furthermore, *E. faecalis* is able to produce cytolysin, a lytic compound which exerts activity against a wide range of gram-positive bacteria and eukaryotic cells including leucocytes and epithelial cells [132]. Also, the lack of genes for the biosynthesis of vitamins and cofactors in Ef_FE21 suggests that symbiotic functions are underrepresented in these bacteria (Fig. 7).

## Conclusion

This study reveals remarkable changes in the microbiota composition of the haematophagous vector *R. prolixus* after exposure to *T. cruzi*, the aetiological agent of Chagas disease, and *T. rangeli*, a vector-pathogenic relative. These alterations broadly reflect the pervasive interactions between host, pathogen and microbiome occurring during colonisation by the parasite and mark the overall importance of the triatomine intestine for the development of *T. cruzi* and, subsequently, the transmission of Chagas disease. Moreover, we were able to reconstruct the genomes of four important symbiotic representatives of *R. prolixus*’ microbiome and assess their metabolic features, one of which has only recently been described. The results of this approach help to understand the triatomine digestive tract as an ecological environment that is shaped and maintained by the influences of the microorganisms living there.

## Supplementary Information

**Additional file 1.** Genome assemblies obtained from NCBI and used for the pangenomic analysis including information on the bacterial strains and GenBank accession numbers.

**Additional file 2.** Genomes obtained from NCBI and used for phylogenomic tree construction of Sp_FE21 including information on GenBank accession numbers.

**Additional file 3. **NCBI SRA identifiers for the raw sequencing data of each sample (BioProject PRJNA744378). The sample names consist of details on the timepoint after exposure (T0-T7), the pathogen used (control, *T. cruzi*, *T. rangeli*) and the intestinal segment (anterior midgut, posterior midgut).

**Additional file 4.** Number of metagenomic read pairs per sample after trimming and the removal of insect reads with bowtie2 (v.2.2.5).

**Additional file 5.** Percentage of reads assigned to a taxonomic group by Kaiju (Menzel et al., 2016) and the proportionate amount of bacterial reads.

**Additional file 6. **A Principle component analysis of the relative abundance of bacterial orders present in the anterior (AM) and posterior midgut (PM) of *R. prolixus*. In total, 81.43% of the overall variance is explained by principle component 1 (PC1, 60.91%), principle component 2 (PC2, 10.57%) and principle component 3 (PC3, 9.95%). B Alpha diversity and species evenness of *T. cruzi*- and *T. rangeli*-exposed samples. AM, anterior midgut; PM, posterior midgut; t, timepoint after exposure.

**Additional file 7.** Shannon and Pielou indices for each sample describing alpha diversity and species evenness, respectively.

**Additional file 8.** Functional modules and genes present in the reconstructed MAGs based on KEGG metabolic pathways. Module definitions, the corresponding hits in the MAGs (kofam_hits_in_module) and module completeness are given.

## Data Availability

The metagenomic sequencing data analysed during the current study are available in the NCBI Sequence Read Archive (SRA) under BioProject accession number PRJNA744378 (https://www.ncbi.nlm.nih.gov/bioproject/744378). Metagenome-assembled genomes have been deposited in NCBI BioSample and are available under the accessions SAMN20089395, SAMN20089396, SAMN20089397 and SAMN20089398.

## Abbreviations

AM: Anterior midgut
COGs: Clusters of orthologous groups
FBS: Foetal bovine serum
GC-content: Guanine-cytosine-content
KEGG: Kyoto Encyclopedia of Genes and Genomes
LIT: Liver-infusion tryptose
LPS: Lipopolysaccharide
MAG: Metagenome-assembled genome
NRPSs: Nonribosomal peptide synthetases
PA: Phosphatidic acid
PCA: Principle component analysis
PE: Phosphatidylethanolamine
PKSs: Polyketide synthases
PKs: Polyketides
PM: Posterior midgut
SCG: Single-copy core gene
