# Supplementary figures and images for: Exposure to Trypanosoma parasites induces changes in the microbiome of the Chagas disease vector Rhodnius prolixus
Source: Microbiome. 2022 Mar 10;10:45. doi: 10.1186/s40168-022-01240-z (PMC8908696; doi:10.1186/s40168-022-01240-z)

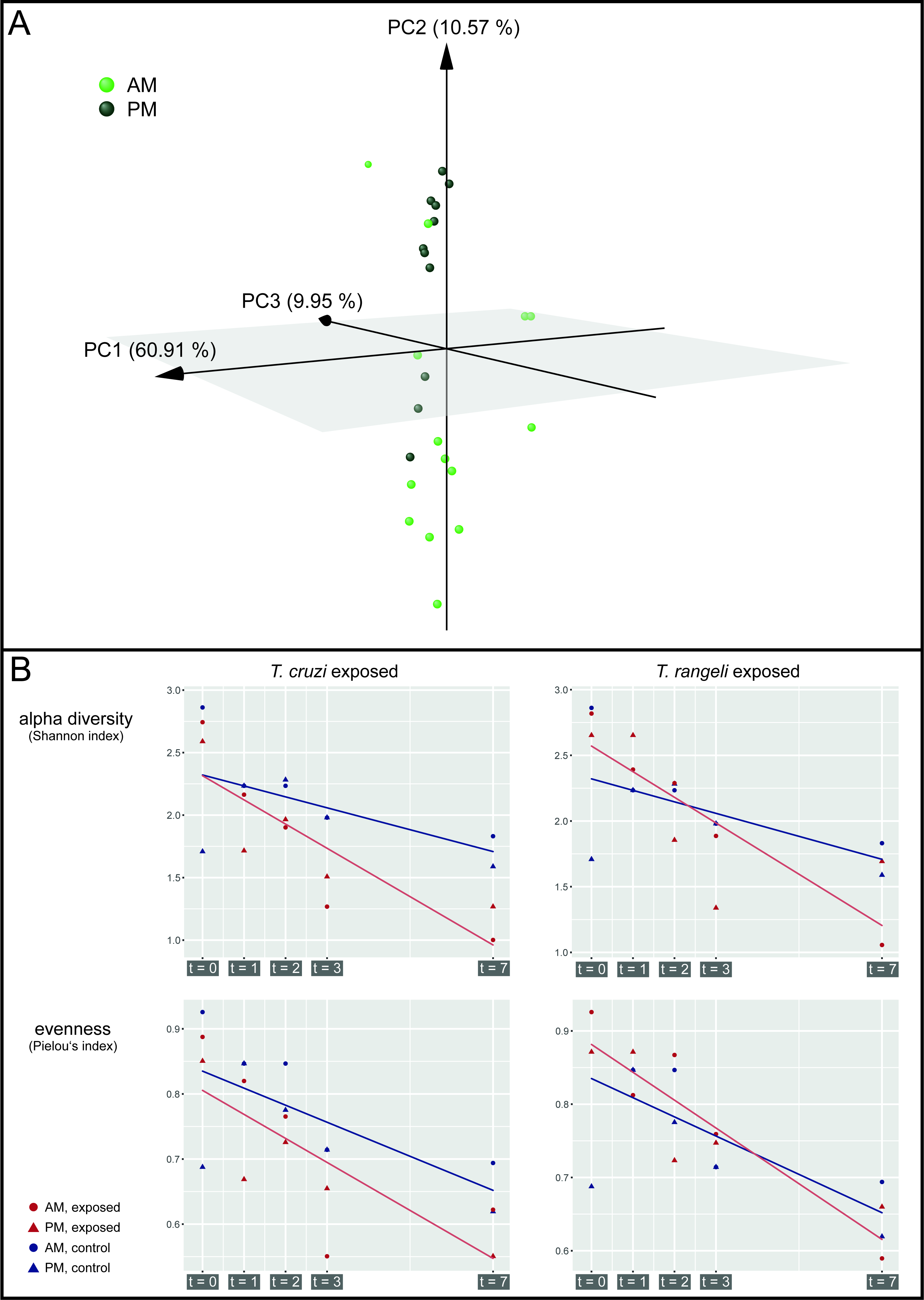

Supplement: Supplementary file 7 — Additional file 6. A Principle component analysis of the relative abundance of bacterial orders present in the anterior (AM) and posterior midgut (PM) of R. prolixus. In total, 81.43% of the overall variance is explained by principle component 1 (PC1, 60.91%), principle component 2 (PC2, 10.57%) and principle component 3 (PC3, 9.95%). B Alpha diversity and species evenness of T. cruzi- and T. rangeli-exposed samples. AM, anterior midgut; PM, posterior midgut; t, timepoint after exposure. [file 40168_2022_1240_MOESM6_ESM.tif]
